# Adhesion G-Protein Coupled Receptors in Neurological and Psychiatric Disorders

**DOI:** 10.20900/jpbs.20250017

**Published:** 2025-11-24

**Authors:** Brandon H. Lee, Christina M. Meyer, David J. Speca, Elva Díaz

**Affiliations:** 1 Department of Pharmacology, School of Medicine, University of California, Davis; 2 Neuroscience Graduate Program, University of California, Davis; 3 Biochemistry, Molecular, Cellular, and Developmental Biology Graduate Program, University of California, Davis

**Keywords:** Receptors, G-protein-coupled, Nervous system disease, Mental disorders, Brain, Neurodevelopment, Synapses

## Abstract

The adhesion G-protein coupled receptors (aGPCRs) are a family of 33 G-protein receptors consisting of ADGRA1–3, ADGRB1–3, ADGRC1–3, ADGRD1–2, ADGRE1–5, ADGRF1–5, ADGRG1–7, ADGRL1–4, and ADGRV1. Recent studies have unveiled the role of aGPCRs in numerous brain functions, including in neurodevelopment, synapse formation and maintenance, establishment of the blood-brain barrier, and myelination. Further, dysfunction of aGPCRs have been associated with disorders such as gliomas, depression, and epilepsy, among many others. Herein, we review generalized properties of aGPCRs, their brain-specific expression, associations with neurological and psychiatric diseases, and potential as future pharmacological targets.

## INTRODUCTION

The adhesion G-protein coupled receptors (aGPCRs) are a family of GPCRs with diverse functions. Despite the prevalence of many aGPCRs in brain tissue, most remain understudied in the context of the nervous system. The aGPCRs are comprised of 33 proteins, organized into nine subfamilies: ADGRA1–3, ADGRB1–3, ADGRC1–3, ADGRD1–2, ADGRE1–5, ADGRF1–5, ADGRG1–7, ADGRL1–4, and ADGRV1 [1]. Understanding the functionality of aGPCRs is critical in developing new pharmacological therapies, as 36% of all approved drugs target GPCRs [2]. In this review, we provide a brief overview of currently understood structural properties and signaling modalities of aGPCRs. We then consider roles for aGPCRs in the context of nervous system functions such as neurodevelopment, synapse modulation, brain vascularization, and myelination. Further, we discuss neurological and psychiatric disorders that arise from aGPCR dysfunction and their capabilities as drug targets.

## METHODS

Searches on PubMed were made for each individual aGPCR using all alternative names. As examples, a search for ADGRG1 included “adgrg1 OR gpr56” and a search for ADGRV1 included “adgrv1 OR vlgr1 OR gpr98”. Primary articles related to structure, potential signaling pathways, nervous system function, and associated neurological or psychiatric disorders were reviewed. Particular focus was given to papers published after 2019 to build upon existing aGPCR reviews [3,4].

For the purposes of this review, the aGPCRs will herein be referred to using the nomenclature established by the aGPCR Consortium and International Union of Basic and Clinical Pharmacology [1]. Alternative names are also provided in Table 1. Gene and protein nomenclature in this review follow conventions for the species of interest. When a specific species is not discussed, human nomenclature is used by default.

To understand the tissue-specific and brain-specific expressions of aGPCRs, we analyzed datasets from the Human Protein Atlas and the Allen Brain Atlas. Each aGPCR and their alternative names were queried in the Human Protein Atlas to identify tissue specificity, highest brain region expression, expression cluster, brain expression cluster, and single cell type specificity [5]. The Human Multiple Cortical Areas SMART-Seq trimmed-means dataset from the Allen Brain Atlas was used to identify brain-specific aGPCR expression [6]. Cell types were determined and categorized following the taxonomy provided by the Allen Brain Atlas dataset.

## OVERVIEW OF ADHESION G-PROTEIN COUPLED RECEPTORS

**Figure 1.**
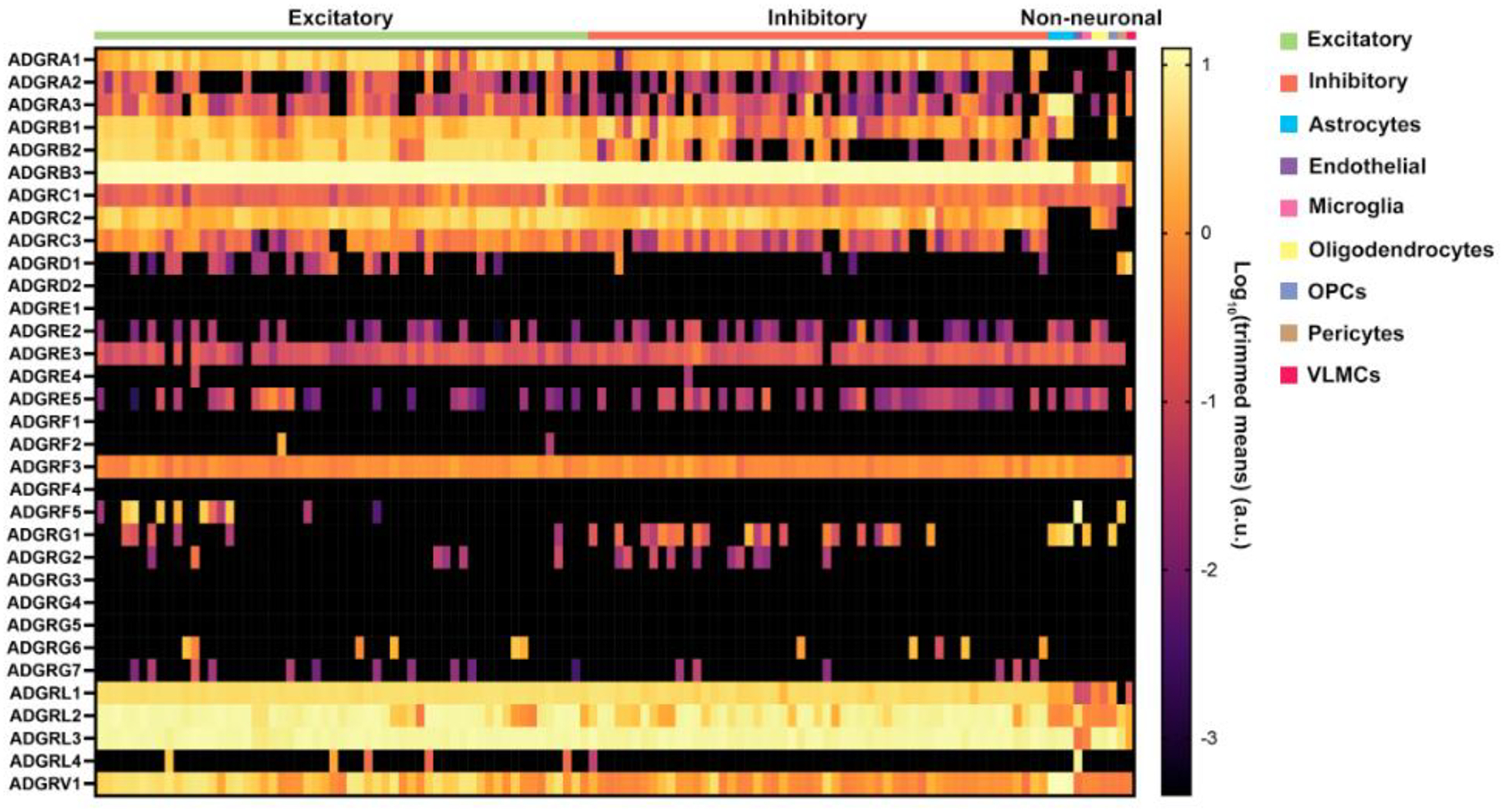
Expression of aGPCRs in nonneuronal and neuronal cells from human cortical regions. Data were indexed from the Allen Brain Atlas Human Multiple Cortical Areas SMART-Seq dataset, which includes transcriptomes from single-nuclei in the middle temporal gyrus, anterior cingulate cortex, primary visual cortex, primary motor cortex, primary somatosensory cortex, and primary auditory cortex [6]. High expression in multiple cell types was observed for ADGRA1–3, ADGRB1–3, ADGRC1–3, ADGRF3, ADGRL1–3, and ADGRV1. Moderate to low cell-specific expression was detected for ADGRD1–5, ADGRF2, ADGRF3, ADGRF5, ADGRG1, ADGRG2, ADGRG6, ADGRG7, and ADGRL4. Expression of ADGRD2, ADGRE1, ADGRF1, ADGRF4, ADGRG3, ADGRG4, and ADGRG5 was not detected in any cell type. Data are represented as log_10_ (trimmed means).

G protein-coupled receptors (GPCRs) are a large superfamily of over 800 described signal transduction-inducing membrane proteins [7] defined by the highly structurally conserved seven-transmembrane pass (7TM) helical structure [8]. GPCRs are ubiquitous in eukaryotes [9], with functional roles in many critical physiological contexts [10]. The GPCR superfamily is grouped into five major families in vertebrates: Glutamate, Rhodopsin, Frizzled/Taste2, Secretin, and Adhesion [11]. aGPCRs expressions vary throughout the body and brain, which are summarized in Table 1 and Figure 1.

aGPCRs are the second largest family of GPCR, with 31 described members in mice and 33 described members in humans [12]. Unlike other GPCR superfamilies, aGPCRs interact with other proteins for activation; most of these proteins are cell membrane-anchored, extracellularly secreted, or in the extracellular matrix. Like other GPCRs, aGPCRs also contain intracellular domains that can recruit protein scaffolds [13], G proteins for signal transduction [14,15], and proteins involved in non-G protein dependent signal transduction cascades, such as β-arrestin, Rac, Rho, and Wnt/β-catenin [4].

**Figure 2.**
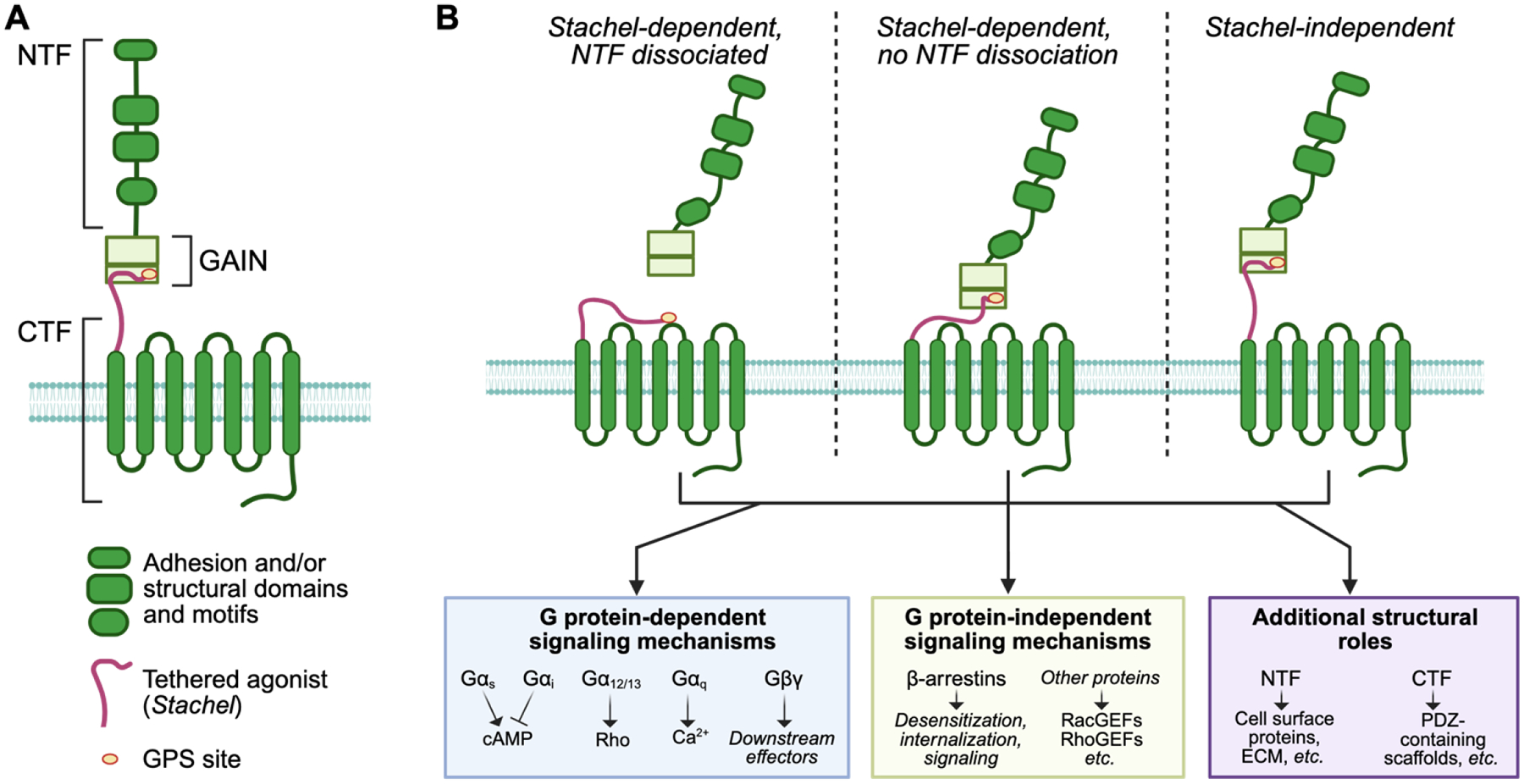
Basic aGPCR structure schematic and activation models. **(A)** The basic structure of all aGPCRs, highlighting the adhesive NTF, the GAIN domain, GPS site and tethered agonist (*Stachel*) sequence, and CTF with the 7TM region and intracellular tail. Individual aGPCRs vary in each of these regions, though some structures, such as the GAIN domain, are highly conserved. **(B)** Models for aGPCR functionality, including G protein-dependent and G protein-independent signaling and structural recruiters and/or stabilizers. All pathways and other roles listed have been identified as functionally relevant in one or more aGPCRs. Figure created using Biorender.

**Figure 3.**
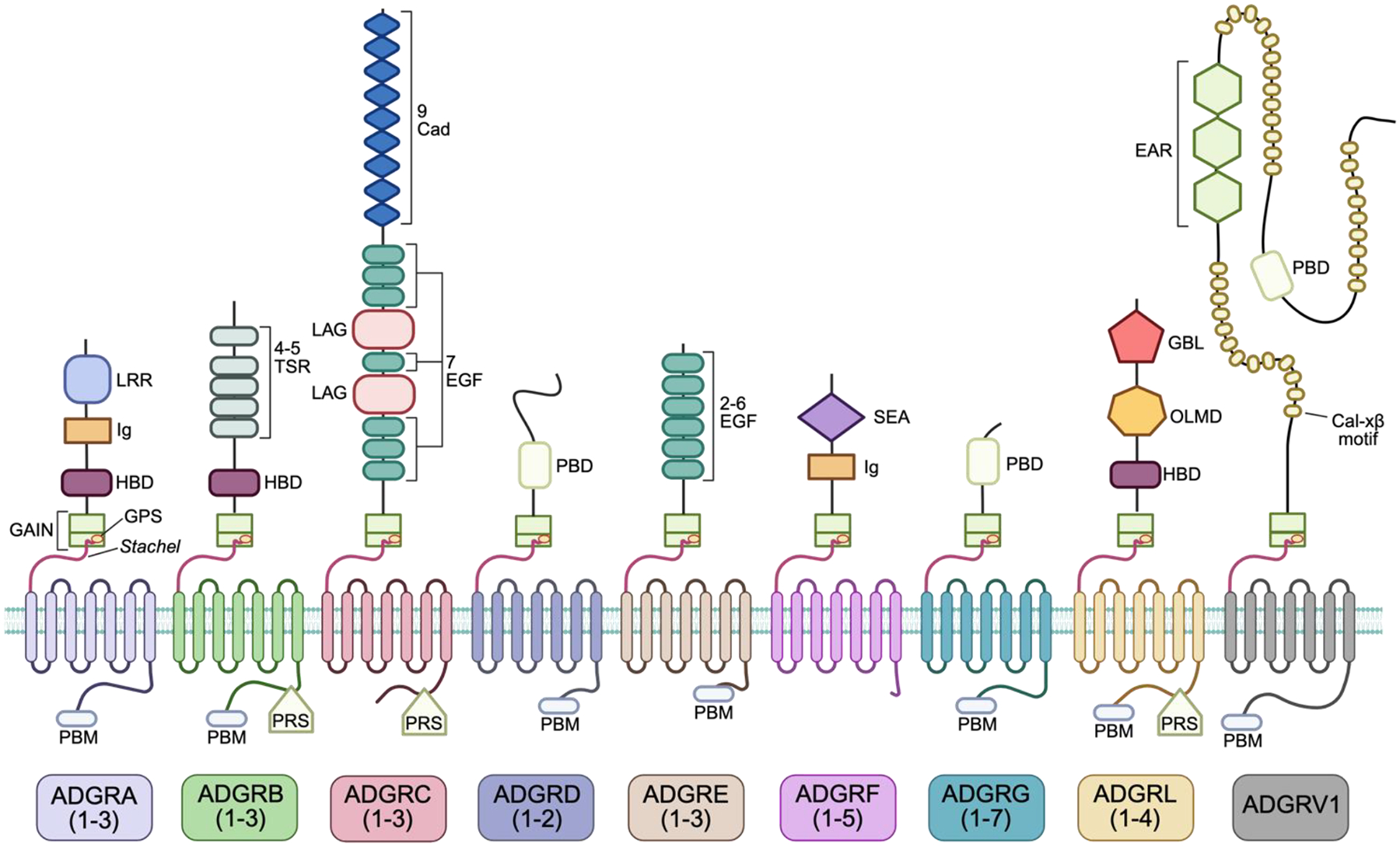
aGPCR subfamily structures. Simplified schematic of aGPCR subfamily extra- and intracellular motifs. aGPCRs are subdivided into nine subfamilies, each beginning with the prefix “ADGR-.” Each family is defined by the presence of a specific combination of adhesion domains or motifs along the NTF, though some aGPCR subfamilies exhibit more diversity in the combination of domains between subfamily members than others. Structures between related domains, such as the pentraxin-binding domain (PDB), may also differ between aGPCR subfamilies. See related Table 2 for more details on presence and absence of domains and motifs within and between aGPCR subfamilies.

However, despite their numbers and high expression across a variety of tissues, aGPCRs remain the least characterized GPCR superfamily; many aGPCRs are orphan receptors with limited understanding of downstream signaling pathways (Table 2, Figure 2, Figure 3) [16]. Additionally, no specific small molecule ligands have been identified for a majority of aGPCRs [17], and those that exist target only the ADGRG subfamily with low specificity [18]. aGPCRs more broadly also lack the microscale activation switch – a structural state where residues form contacting interactions that are found in both the active and inactive state. Instead, they can rapidly enter active state contacts upon binding to inverse agonists [19]– common to all other GPCR superfamilies [20], further obfuscating models of aGPCR activation. Similarly, the aGPCRs display remarkable selectivity and diversity, both between and within subfamilies; for example, though some aGPCR subfamilies and individual subfamily members contain many of the same adhesion domains, structural changes due to the presence of other domains and post-translational modifications significantly changes their adhesion properties and, presumably, activation mechanisms related to adhesion [4]. Thus, research into aGPCRs offer many opportunities to study both these unique receptors and the mechanisms of structural divergence between GPCR superfamilies.

### aGPCR Structure and Properties

The overall structure of all aGPCRs is divided into the N-terminal fragment (NTF), the GPCR autoproteolysis-inducing (GAIN) domain, and the C-terminal fragment (CTF) (Figure 2A). The NTF typically is described as containing a large majority of the extracellular domains. The CTF typically is described as containing the 7TM region and all intracellular domains. The GAIN domain is sometimes considered as split between the NTF and CTF at the site of autoproteolysis, though some reviews have considered it a distinct region. For the purposes of this review, the GAIN will be discussed as a separate region. Recent advances in cryogenic electron microscopy (cryo-EM) have expanded our understanding of aGPCR structures and functionality, particularly at the GAIN and 7TM regions, while continued functional research continues to further our understanding of aGPCR binding and signaling.

#### NTF Structure and Properties

All aGPCRs NTFs contain the adhesion domains that give the aGPCRs their namesake and account for the majority of each aGPCR’s molecular weight [4]. Extensive alternative splicing [32] can also lead to large variations in aGPCR size and function [33]. aGPCRs use these adhesion domains to bind to other extracellular proteins, which can activate or inhibit receptor functionality.

Each aGPCR subfamily is typically characterized by variable numbers of structurally well-defined and modular adhesion domains [4]. The presence of these domains within subfamilies, as well as extensive post-translational modifications [34], allows for binding to a diverse set of cell surface and extracellular matrix proteins [32,35,36].

While the presence and order of adhesion domains are typically specific to each aGPCR subfamily, other structures within the NTF are shared between groups. One example is the complement C1r/C1s, Uegf, Bmp1 (CUB) and CUB-like adhesion domains on the ADGRB subfamily members, as well as on ADGRG6/GPR126. Additional motifs seem subfamily specific, such as the sea urchin sperm protein/enterokinase/agrin (SEA) module in ADGRFs and the Calxβ motif in ADGRV1. These structures may also play roles in modifying adhesion properties through cleavage at the SEA [37] and calcium binding at Calxβ motifs [31]. However, the most notable instance of this interfamily domain sharing is the approximately 70-residue hormone-binding domain (HBD), which is present in the ADGRA and ADGRB subfamilies. Though the name implies hormone binding, no hormone has been found to bind the hormone domain thus far [38]. Some structural data suggests the HBD remains rigid [39], which may give the NTF different structural conformations, though more research must be conducted in this area to conclusively determine the function of the HBD domain.

#### The GAIN domain

All aGPCRs except for ADGRA1/GPR123 contain the GAIN domain; consequentially, the GAIN domain is considered a critical feature of aGPCRs. This highly conserved structure was among the first structural characteristics of aGPCRs to be resolved via X-ray crystallography [39] and remains a domain of high interest within aGPCRs due to its importance for aGPCR signaling and functionality.

The GAIN domain is typically split between the NTF and CTF at the GPCR proteolysis site (GPS). The GPS is an autoproteolysis site, wherein self-sufficient protease activity cleaves (denoted by ‘/’) at a highly conserved H or R-L/T or S consensus sequence by way of nucleophilic attack [40,41]. However, the NTF remains non-covalently associated with the CTF across the cleavage site due to protein refolding [42]. Autoproteolysis may be regulated by N-linked glycosylation events and other posttranslational modifications [43], opening additional complexities to existing models of aGPCR cleavage.

After proteolysis, the GAIN domain develops unique structural properties. The portion upstream of the GPS is α-helix rich and contains a C-X-C or X-X-C sequence 6–9 residues upstream from the GPS site [42]. Immediately downstream of the GPS is a β-strand encoded by the highly conserved sequence X-F-A-V-L-M, also known as the tethered agonist or *Stachel* sequence [18,44]. This *Stachel* sequence is modeled to facilitate receptor activation [18,44], though many publications suggests that not all aGPCRs undergo autoproteolysis [45] and/or *Stachel*-promoted activation [46–48] under in vitro and physiological conditions [49].

#### CTF Structure and Properties

The 7TM domain is present in all aGPCRs [50]. To that end, the aGPCR GAIN and 7TM domains are most often modeled as facilitating receptor activation together, with the GAIN domain releasing the *Stachel* tethered agonist, which can subsequently act analogously to the small peptide ligands of members of the Secretin-GPCR superfamily, like the glucagon-like peptide-1 receptor [39,50]. Cryo-EM was utilized to identify interactions between the *Stachel* and 7TM region in receptors modeled to undergo NTF dissociation-dependent tethered agonist activation. Both ADGRD1/GPR133 and ADGRG2/GPR144 undergo a structural reorganization, forming a binding site for the tethered agonist sequence within the 7TM formed from transmembrane domains 1, 4, and 5 [51]. Both structures were also isolated alongside Gαs, Gβ, and G*γ*, further demonstrating that tethered agonism allows for the recruitment of G protein complexes [51]. Additional structures of cleaved ADGRG1/GPR56 and ADGRL3/Latrophilin3 bound to Gα_13_, Gβ, and G*γ* have also been reported [52]. However, cryo-EM studies have also identified cleavage-deficient variants of ADGRF1/GPR110 that still have the tethered agonist sequence positioned within the 7TM pocket [53], opening more questions as to how the GAIN domain may organize itself in autoproteolytically processed and non-processed receptors. Thus, two models exist for tethered agonist-dependent activation: one wherein GAIN-autoproteolysis allows for dissociation between the NTF and CTF at the GPS site, and one wherein the tethered agonist can regulate receptor signaling with or without GAIN-autoproteolysis [54]. Both models rely on mechanical forces from binding interactions at the NTF [55], positing aGPCRs as both mechanosensitive receptors and adhesive-dependent receptors.

The 7TM domain also contains other regions that may have functional importance, such as the accompanying extracellular (ECLs) and intracellular loops (ICLs), and the intracellular C-terminal tail. The function of the ECLs and ICLs, known to be critical for extracellular ligand binding [56] and G protein and β-arrestin signaling and regulation [57,58] in other GPCRs, remains unclear; the ECLs are particularly poorly characterized in aGPCRs, and do not appear to bind small molecule ligands in the same way as other GPCRs. Cryo-EM structures have also shown that the ICLs of ADGRL3 are critical for G protein coupling [59], suggesting that the ICL regions may have significant structural and functional similarities between aGPCRs and other GPCR superfamilies. However, many aGPCRs have varying lengths of both ICLs and ECLs due to alternative splicing [60], suggesting that signaling may differ due to ICL and ECL changes in different physiological contexts.

The intracellular C-terminal tails of different aGPCR subfamilies vary in length and functionality. Proline-rich regions located in the C-terminal tail of some aGPCRs are potentially capable of forming polyproline helices, which may affect intracellular binding and signaling cascades [61], though these sequences have not been extensively studied in aGPCRs. Many aGPCRs also contain a PDZ binding motif that allows for binding interactions to PDZ domain-containing proteins, which play significant roles in the recruitment and anchoring of cell surface receptors in many different tissues [62]. Phosphorylation along the CTF is also common [63], and likely promotes binding of β-arrestins, much like in other canonical GPCRs [64].

#### Summary

Altogether, the complicated structures of aGPCRs reflect the complex roles they play in a variety of cellular and physiological contexts. Current research indicates that many aGPCRs can signal in a *Stachel*-dependent manner, both with and without the dissociation of the NTF at the GPS, as well as in a tethered agonist-independent manner, as a protein scaffold or recruiter of other protein complexes. Furthermore, G protein-dependent and G protein-independent signaling pathways have been identified in *Stachel*-dependent and -independent paradigms for numerous aGPCRs, as well as with and without NTF dissociation. The additional roles of both NTF and CTF motifs that can recruit certain structural scaffolds only add further complexity to these models (see Figure 2B for functional models of aGPCRs). Advances in cryo-EM and structural modeling algorithms have allowed for novel insights into the structure of aGPCRs, although questions around their activation mechanisms remain unanswered. Better models of aGPCR structure and function will be critical for designing targeted therapeutics for a variety of conditions, particularly neurological and psychiatric conditions that may have little to no other viable treatment options. A summary of the neurological and psychiatric disorders associated with aGPCRs is provided in Table 3 and expanded upon in the next sections.

## ADHESION G-PROTEIN COUPLED RECEPTORS IN NEUROLOGICAL AND PSYCHIATRIC DISORDERS

### ADGRAs

#### ADGRA1

ADGRA1 is expressed primarily in the cortex, thalamus, hypothalamus, and hippocampus, with moderate expression in the amygdala, hypothalamus, inferior olive, and spinal cord [66] and has been localized to the postsynaptic fraction [67]. Loss of *Adgra1* in male mice results in increased anxiety-like behaviors [68], increased spine density [68], upregulation of PSD-95 [68], and hypothalamic misfunction resulting in abnormal energy expenditure and thermogenesis [69]. A recent preprint also suggests that ADGRA1 is important in proper development of hippocampal inhibitory connections, where loss of ADGRA1 in parvalbumin-positive (PV+) and somatostatin-positive inhibitory interneurons results in decreased amplitudes of evoked inhibitory synaptic currents and subsequent impairment of Pavlovian fear conditioning in mice [70]. Further, an analysis of the Cancer Glioma Atlas revealed that expression of the anti-sense long non-coding RNA variant of *ADGRA1*, *ADGRA1-AS1,* was associated with better prognosis for glioma patients [71]. Together, these data suggest that ADGRA1 could be involved in establishment of synaptic circuitry and a potential therapeutic target for anxiety, metabolic disorders, and glioma.

#### ADGRA2

Of the ADGRA subfamily, ADGRA2 is the most extensively studied. ADGRA2 is a proangiogenic receptor expressed in endothelial cells and pericytes, whose activity is critical for the development of the blood-brain barrier (BBB) [72–75]. ADGRA2 modulates angiogenesis via β-catenin signaling through complex signaling interactions with Wnt7a / 7b, the GPI-anchored protein Reck, the Frizzled receptor, and Dishevelled [72–79]. In the absence of ADGRA2, Reck binds Wnt7a / 7b, preventing activation of Frizzled receptors by Wnt [15]. ADGRA2 binds Reck extracellularly, bringing Reck-bound Wnt7a / 7b into proximity of the intracellularly bound Frizzled receptor [74,77]. In zebrafish, Dishevelled is a required adaptor between ADGRA2 and the Frizzled receptor, but human and mouse variants of Adgra2 do not contain Dishevelled binding sites in their intracellular domains [72,77]. Activation of the Frizzled receptor by binding of WNT7a / 7b triggers downstream pathways that regulate β-catenin [74,77,79,80]. Thus, disruption of *Adgra2* activity leads to cerebral vascularization defects as well as impaired formation of dorsal root ganglia, leading to embryonic lethality [73–75,77,79,81–84]. Mutations in *ADGRA2* have been identified in patients that associated with polymicrogyria [85] and malformation of the cerebellum, spinal cord and cerebral cortex [86]. Interestingly, these mutations led to bifrontal polymicrogyria similar to deleterious *ADGRG1* mutations [86], but not vascular abnormalities as expected with ADGRA2’s role in the BBB. However, another study did identify 3 *ADGRA2* variants in patients associated with reduced risk of developing brain arteriovenous malformation [87].

Aside from its role in development, ADGRA2 is also required for effective response to disruptions of the BBB in adults. Models of ischemia have associated loss of *Adgra2* with additional devastating defects. Oxygen deprivation increases *Adgra2* expression in pericytes, where it localizes in filopodia to modulate cell polarity and cell adhesion through interactions with the ELMO / DOCK complex and intersectins [88,89]. ADGRA2 promotes ELMO phosphorylation, leading to activation of CDC42 and Rac1 GTPases that are imperative for polarization of cells towards injury sites [89]. In response to ischemic stroke, mice with conditional knockout of *Adgra2* in endothelial cells exhibit increased breakdown of the BBB, microvascular hemorrhage, and lower overall survival [82]. Conversely, overexpression of *Adgra2* leads to increased pro-inflammatory signaling and pyroptosis, which are also associated with decreased survival rates [90]. Even a truncated fragment of the ADGRA2 NTF can improve cognitive function in mice following bilateral common carotid artery occlusion by promoting cell migration and extracellular matrix adhesion [91]. Further, an analysis of nine neuroinvasive viruses identified ADGRA2 as a potential host protein containing viral protease cleavage sites [92]. This suggests that cleavage of ADGRA2 may assist viruses in bypassing the BBB. Together, these data suggest that careful regulation of *Adgra2* is required for proper modulation of ischemic injury.

ADGRA2 also plays a role in nervous system cancers. ADGRA2 binds ch-TOG to promote microtubule assembly and regulate the cell cycle [93]. Intriguingly, upregulation or downregulation of *Adgra2* decreases cell proliferation in glioblastoma cells [93]. Similarly, silencing of *Adgra2 in vitro* inhibits tumor growth and blood vessel formation [94], while conditional knockout of *Adgra2 in vivo* amplifies intratumoral hemorrhage and edema [82]. These studies again suggest that balanced levels of ADGRA2 expression are required for suppression of gliomas. Additionally, high ADGRA2 expression has been associated with poor prognoses in patients in lung adenocarcinoma due to its role in promoting brain metastases [95]. The activation of WNT7a / 7b mediated β-catenin signaling promotes trans-endothelial migration in vascular pericytes, leading to the spread of cancer cells to the brain [95]. Finally, studies have also observed that patients with rectal neuroendocrine carcinomas are associated with mutations in ADGRA2 [96].

#### ADGRA3

Work on the involvement of ADGRA3 in neuropsychiatric functions remains limited. ADGRA3 is expressed in regions of the cortex, hypothalamus, and choroid plexus [97]. One study has illustrated that *Adgra3* is specifically upregulated in the choroid plexus following traumatic brain injury (TBI) [97]. However, the mechanisms by which this upregulation occurs, and subsequent downstream effects remain unclear. Other studies have also suggested a role of ADGRA3 in development. ADGRA3 is differentially expressed throughout the formation of the cochlea but is not required for its development or functional hearing [98]. Further, ADGRA3 recruits Dishevelled to the cell membrane during gastrulation to regulate Wnt/PCP signaling [99]. This, in turn, drives convergence and extension movements critical for proper establishment of developmental axes. Overexpression of *Adgra3* disrupts these movements and loss of *Adgra3* results in enhanced defects of PCP mutants, including in neuronal migration [99]. Interestingly, the ADGRA3 LRR domain is sufficient for proper trafficking of ADGRA2 in a chimeric protein [76] but ADGRA3 does not signal with WNT7A / B specifically [80]. However, the similarities to ADGRA2 in modulation of Wnt signaling and cell polarity suggest that ADGRA3 could be a critical and distinct regulator of development.

### ADGRBs

#### ADGRB1

Numerous functions of ADGRB1 have been identified in the nervous system, including in synaptic development, angiogenesis, and neuroimmune function. ADGRB1 modulates dendritic and axonal arborization in a RhoA-dependent fashion. Loss of *Adgrb1* leads to low RhoA activity and triggers dendritic outgrowth, while overexpression leads to high RhoA activity and dendritic retractions [34,100]. In dendritic spines, ADGRB1 interacts with postsynaptic proteins such as PSD-95 [101,102] to regulate spine density, spine length, and spine diameter [103–105]. This modulation occurs via an interaction between ADGRB1 and PAR3, which localizes the PAR3 / TIAM1 complex to dendritic spines to activate RAC1 and induce cytoskeletal remodeling [104,105]. Further, ADGRB1 has been shown to bind RTN4Rs [34] and complement component 1q [106] to mediate additional synaptic roles. Functionally, loss of *Adgrb1* decreases the frequency of miniature excitatory post-synaptic currents and impairs long-term potentiation and long-term depression in neurons of the hippocampus [101,105]. Reduced expression of PSD-95 in *Adgrb1* knockout mice, likely due to increased PSD-95 polyubiquitination, indicates a disruption of the organization of postsynaptic proteins in the absence of ADGRB1 [101]. Together, these alterations result in social deficits and increased susceptibility to seizure in mice [103]. Interestingly, in humans, *de novo* mutations in *ADGRB1* have been associated with autism spectrum disorder (ASD) [107]. Recently, ADGRB1 has also been shown to be necessary for fully functional hearing, being involved in the localization of AMPA receptors in the postsynaptic density of type I spiral ganglion cells [108].

ADGRB1 has been identified in multiple brain cancers. Studies have illustrated a downregulation of ADGRB1 in medulloblastoma [109,110], glioblastoma [111–113], astrocytoma [114], and lung adenocarcinoma brain metastases [115]. Decreased expression of ADGRB1 in brain cancers is thought to occur through extensive methylation of the *ADGRB1* locus by methyl-CpG binding domain protein 2 (MDB2) and Enhancer of zeste homolog 2 (EZH2) [109,110,112,116]. ADGRB1 also stabilizes p53 levels by removing the E3 ubiquitin-protein ligase Mdm2 from the nucleus [109]. This dual function makes ADGRB1 an interesting potential target for treatment of these cancers. Excitingly, ADGRB1 overexpression in medulloblastoma and glioblastoma by blocking MDB2 and EZH2, or ADGRB1 injection inhibits tumor angiogenesis [111,112] and stabilizes p53 [109,110], leading to increased odds of survival in mice. Relevant to public health, increased methylation of *ADGRB1* is also present in neonates with mothers exposed to electronic waste and heavy metals [117,118].

Further, ADGRB1 has been shown to be involved in macrophage and astrocyte function through binding of phosphatidylserine. Upon binding phosphatidylserine on apoptotic cells, ADGRB1 interacts with the ELMO / Dock180 complex to recruit Rac-GEF complexes and promote engulfment of apoptotic cells [119–121]. This interaction also mediates recognition of surface lipopolysaccharide and engulfment of gram-negative bacteria [122]. Reduction of ADGRB1 also leads to impaired formation of the phagocytic cup, leading to reduced branch retraction and bacteria clearance efficiency [123]. However, there is some controversary whether ADGRB1 is endogenously expressed in macrophages, or if these effects are attributed to ADGRB1 expression in other phagocytes [124].

#### ADGRB2

ADGRB2 is primarily expressed in the cerebral cortex, hippocampus, cerebellum, and brainstem nuclei and is specifically enriched at postsynaptic sites [125,126]. Loss of *Adgrb2* results in decreased density of glutamatergic synapses and mature mushroom spines without affecting GABAergic synapses [125]. Disruptions in *Adgrb2* have been associated with antidepressive behaviors, increased adult hippocampal neurogenesis, and hyperactivity [127,128]. In one clinical case, a mutation in the C-terminal domain (R1465W) was associated with the development of progressive spastic paraparesis and other neurological symptoms [129]. This mutation resulted in increased constitutive signaling of NTF-cleaved ADGRB2, switching activity from G_αz_ coupled to G_αi_ coupled signaling and disrupted binding to endophilin A1 [129]. Interestingly, recent large-scale exome-wide sequencing analyses and genome-wide association studies have identified ADGRB2 expression to be significantly correlated with depressive symptoms [130], neuroticism [131], and decreased educational attainment [132].

#### ADGRB3

ADGRB3 has been identified as a critical regulator of synapse development in the hippocampus, cerebral cortex, and cerebellum. In mice, loss of *Adgrb3* leads to social deficits [133], smaller brain and body weights [133,134], abnormal energy expenditure [134], and increased susceptibility to seizure [133]. ADGRB3 interacts with synaptic protein complexes ELMO / DOCK180 / RAC1 [135], neuronal pentraxins 1/R [136], and the four component complement 1, Q subcomponent – like (C1QL) proteins [137–139]. In hippocampal neurons, disruption of ADGRB3 leads to defects in dendritic length, branching and density of excitatory synapses [135,137]. Furthermore, in a mouse model of Alzheimer’s disease, microRNA-142–5p is overexpressed, leading to downregulation of hippocampal ADGRB3 expression [140]. When microRNA-142–5p was inhibited, ADGRB3 was upregulated and impairments in spatial learning and memory were reduced [140]. During cerebellar development, C1QL1 in climbing fibers interacts with postsynaptic ADGRB3 on Purkinje cells and loss of either impairs motor learning [141]. This interaction is required for synapse elimination and synaptogenesis to determine a “single winner” climbing fiber that exclusively innervates a Purkinje cell [141–143]. In the basolateral amygdala, C1QL3-containing neurons that project to the medial prefrontal cortex are required for the proper development of implicit association and fear memories [144]. In these neurons, ADGRB3 additionally interacts with C1QL3 and PSD-95 to mediate formation of morphine withdrawal memories [145], making ADGRB3 an appealing target to facilitate recovery from substance use disorders. Further, the projections from the anterior olfactory nucleus also contain C1QL3, which binds postsynaptic ADGRB3 in the olfactory bulb [146]. Loss of C1QL3 or ADGRB3 activity leads to a decrease in the number of synapses from the anterior olfactory nucleus to the olfactory bulb and impairment of learning in social transmission of food preference, without affecting olfactory function [146]. Together, these studies suggest that ADGRB3 is a critical regulator of synaptic development in multiple brain regions and is specifically required for memory-related functions.

ADGRB3 has also been linked to other roles in the nervous system. In the cochlea, ADGRB3 interacts with C1QL proteins and modulates levels of ELMO1 / DOCK180 / RAC1 [147]. Loss of *Adgrb3* leads to high-frequency hearing impairment, thinner pillar cells, and degeneration of hair cells and spiral ganglion neurons in older mice [148]. C1QL1 also promotes differentiation of mature oligodendrocytes, possibly through an interaction with ADGRB3 [149]. After cerebral ischemia, ADGRB3 levels are downregulated [150] and could be involved in C1QL1/4-mediated angiogenesis [151]. Unsurprisingly, ADGRB3 has been implicated in various disorders. Human genetic studies have associated ADGRB3 expression and mutations with anxious temperament [152], taste perception degeneration in Alzheimer’s disease [153], development of Chiari Malformation Type I [154], disorganized symptoms of schizophrenia [155,156], multiple sclerosis [157], predisposition to substance use disorders [158], cerebral and cerebellar atrophy [159], intellectual disability [159], major depressive disorder [155], and ASD [160]. ADGRB3 could also be a marker for large cell neuroendocrine carcinoma [161] and is downregulated in gliomas [150]. Of further clinical relevance, perinatal exposure to selective serotonin reuptake inhibitors alters expression of ADGRB3 in multiple brain regions, subsequently increasing passive stress coping and decreasing sucrose preference [155]. Together, these studies suggest that ADGRB3 could be a powerful therapeutic target for neurological and psychiatric disorders.

### ADGRCs

#### ADGRC1

ADGRC1 is a planar cell polarity protein involved in coordination of cells during neurodevelopment. *ADGRC1* variants have been identified in patients with neural tube-related defects and brain malformations [162–171], partial epilepsy of childhood [172], ischemic stroke [173–175], spina bifida [176], glaucoma [177], familial strabismus [178], Phelan-McDermid syndrome [179], and Parkinson’s disease [180]. Expression of *ADGRC1* has also been associated with glioma [181], cerebral ischemic injury [182], and child behavioral issues [183]. Similarly, loss of functional *Adgrc1* in mice leads to high embryonic mortality [184], neural tube defects [185–187], vestibular dysfunction [184,185,188], and aberrant migration of facial branchiomotor neurons [189–191]. Throughout embryonic development, *Adgrc1* is regulated along the apico-basal axis [46], expressing in the ventricular zone of the neural tube [192]. In this apical region, ADGRC1 determines mediolateral polarity by recruiting Dishevelled-2, which associates with PDZ-RhoGEF through DAAM1 [187]. This complex activates Rho kinases that promote midline convergence of neuroepithelial cells [187]. Failure of this pathway leads to abnormal neural plate morphology and neural tube closure defects [185,186]. Beyond neural tube closure, ADGRC1 interacts with Wnt / PCP proteins to mediate retinoic acid signaling in apical neural progenitor cells [193]. Deficient ADGRC1 dysregulates retinoic acid, triggering self-renewal of progenitors over neurogenesis and leading to cortical hypoplasia [193]. Additionally, ADGRC1 has been associated with dorsal sensory tract morphogenesis [46] and dendrite initiation in granule cells [194] in mice, as well as axon trajectory defects in *C. elegans* [195].

*Adgrc1*-deficient mice that survive past fetal development exhibit vestibular dysfunction. Behaviorally, they exhibit circling behaviors, nystagmus, gaze instability, and impaired vestibular-ocular reflexes [184,188]. This is, in part, due to failure of stereocilia bundles to polarize and align [184]. In the cochlea, ADGRC1 is regulated by Wnt proteins [196] and stabilizes an intracellular signaling complex of two planar polarity proteins, Frizzled 3/6 and Van Gogh-like 1/2 [197]. Disruption of any of these three proteins leads to type II spiral ganglion neuron tuning errors, incorrect innervation of cochlea fibers, and developmental defects in the semi-circular canal cristae [184,197]. These impairments lead to defects in the vestibular system, leading to altered behaviors. Further, *Adgrc1*-deficient mice also exhibit defects in migration of facial branchiomotor neurons. Normally, ADGRC1 suppresses chemoattractant Wnt5a to properly guide facial branchiomotor neurons from rhombomere 4 to rhombomere 6 [189]. Loss of *Adgrc1* leads to improper migration rostrally to rhombomere 3, due to attraction mediated by Wnt5a [189,191]. Thus, ADGRC1 mediates directionality of migration for facial branchiomotor neuron migration [189–191].

#### ADGRC2

*ADGRC2* is also a planar cell polarity protein with distinct functions from *ADGRC1*. *ADGRC2* variants have been associated with neural tube defects [170], idiopathic scoliosis [198], Joubert syndrome [199,200], epilepsy [201], and Alzheimer’s disease [202]. During zebrafish development, *adgrc2* modulates forebrain wiring through an interaction with *frizzled 3* and *van Gogh-like 1/2* [203], initiates facial branchiomotor neuron migration with *adgrc3* [191,204], and regulates axon growth cone guidance together with other planar cell polarity proteins [205]. Further, *Adgrc2* in mice promotes neurite outgrowth [206], ciliogenesis [207,208], Schwann cell proliferation and migration [209], and polarization of reactive astrocytes [210]. The specific impairment of ciliogenesis is a hallmark of Joubert syndrome and also leads to hydrocephalus [208].

Interestingly, multiple functions for *Adgrc2* have also been observed in the adult brain. Inhibition of *Adgrc2* in adult mice leads to motor learning deficits due to disruption of layer V pyramidal neurons *→* dorsal striatum projections [211]. This loss impairs spine formation, leading to decreased excitatory synapse density and signaling and increased inhibitory synapse density and signaling [211]. This disruption in excitatory / inhibitory balance could explain the epileptogenesis observed in humans. Further, loss of *Adgrc2* in dorsal CA1 pyramidal neurons leads to defective social memory, due to impairment of NMDAR-mediated synaptic transmission [212]. Conversely, in motor neurons, *Adgrc2* negatively regulates axon regeneration and fasciculation, impairing neurite and growth cone outgrowth [213,214]. This discrepancy suggests that *Adgrc2* may function bidirectionally in neurite growth depending on a cell-specific context. Beyond these functions, *Adgrc2* expression has been found to be related to glioblastoma progression [215], herbicide exposure [207], and seizures [201,216].

#### ADGRC3

ADGRC3 is an essential regulator of axon guidance. *ADGRC3* has been associated with Tourette’s syndrome [217–220], epilepsy [221,222], Rubinstein-Taybi syndrome and schizophrenia [223], perineural invasion of oral squamous cell carcinoma [224], migraine and stroke [225], central hypotonia [226], and neuroendocrine cancers [227–229]. Loss of *Adgrc3* disrupts numerous axon tracts, including in the acoustic startle hindbrain circuit [230], internal capsule [231], subventricular zone *→* olfactory bulb projections [232], rubrospinal and corticospinal tracts [233–236], motor neurons [237], fine sensory fibers [238], globus pallidus [239], hippocampal architecture [240], thalamocortical circuits [241–243], GABAergic retinal circuits [244], and neocortical interneurons [245]. This modulation relies on an interaction with *Frizzled3* [242,246–248] to promote Wnt-mediated outgrowth [248] and establish pioneer neuron scaffolds [242]. In the hindlimb, *Adgrc3* and *Frizzled3* have been observed to function through modulation of chemoattractive EphA-ephrinA reverse signaling [237]. This interaction mediates *Jag1* expression in response to *Wnt7* and *Notch* signaling to regulate neurogenesis in immature cortical neurons [249]. Despite ADGRC3 mediation of broad and diverse axonal path finding, the exact mechanisms by which it acts requires further investigation.

Specific disease-related interactions of ADGRC3 have recently been identified in neuromodulatory circuits. *Adgrc3* determines guidance of dopaminergic and monoaminergic neurons [250–252]. This function is also reliant on an interaction with *Frizzled3*, along with Wnt5a chemoattraction of serotonin neurons and Wnt7b chemoattraction of dopaminergic neurons [250]. In mice, loss of *Adgrc3* resulted in Tic-related behaviors, recapitulating symptoms of Tourette’s syndrome [251,252]. This loss is specifically associated with dysregulation of D_3_ dopamine receptors [251], which subsequently results in impaired motor function, dopaminergic signaling, and reward learning [251,252]. Interestingly, *Adgrc2* and *Adgrc3* expression in basolateral amygdala projecting neurons from the infralimbic prefrontal cortex is required for restoration of glutamatergic synapses and antidepressant response to ketamine treatment in mice [253]. Further, oligomeric β-amyloid has been shown to bind *Adgrc3*, recruiting *Van Gogh-like 2* to promote disassembly of synapses and subsequent synapse degeneration [254]. These studies suggest that *Adgrc3* functions in the adult brain and may contribute to disease etiology beyond developmental defects.

### ADGRDs

#### ADGRD1 and ADGRD2

Studies on the role of ADGRD1 and ADGRD2 in the brain are limited. ADGRD2 is primarily expressed in seminal vesicles and is not detected in cortical regions, suggesting that it does not play a role in nervous system function (Table 1, Figure 1). However, ADGRD1 has been identified as a potential protumorigenic protein in gliomas. ADGRD1 expression is sparse in non-cancerous brain tissue but is upregulated in the progression of gliomas [255]. The presence of ADGRD1 promotes tumor initiation and growth in a hypoxia-dependent manner, with knockdown of *Adgrd1* eliminating tumor initiation in mice injected with human glioblastoma cells [256,257]. High expression of *ADGRD1* is correlated with glioma severity, as well as poor prognoses and reduced survival [255,257,258]. Efforts to downregulate *ADGRD1* expression have included targeting the NTF-cleavage-dependent activity [259], *ADGRD1* binding partners extended synaptotagmin 1 [260] and PTK7 [261], as well as downstream microRNA miR-106a-5p [258]. The prevalence and necessity of ADGRD1 in glioma development, as well as multiple identified modulators, make ADGRD1 an appealing candidate for further studies in glioma treatment.

### ADGREs

#### ADGRE1–5

The ADGRE subfamily is primarily involved in the immune system, with relatively low expression in the brain (Table 1, Figure 1). The extensive roles of ADGREs in regulating inflammation have been reviewed previously [4,262]. Relevant to neurological disorders, *ADGRE1* single nucleotide polymorphisms (SNPs) have been identified in African children with falciparum malaria that increase risk of developing complex malaria-associated seizures, which include repetitive and coma-inducing seizures [263]. Further, a genome-wide association study in Korean children associated many SNPs in *ADGRE1* with high-risk, MYCN-amplified neuroblastoma [264]. ADGRE5 expression levels have also been associated with invasion of glioma cells [265,266]. Together, these results suggest possible indirect roles for ADGRE1 and other ADGREs in neurological and psychiatric disorders in the regulation of the neuroimmune function.

### ADGRFs

#### ADGRF1

ADGRF1 regulates nervous system development as a receptor for the ligand synaptamide. Expression of ADGRF1 is high in fetal brain tissue but is minimal after birth [267] (Figure 1). Synaptamide promotes neurogenesis and synaptogenesis [267] in an ADGRF1-dependent manner through binding to the GAIN domain [268]. This binding upregulates cAMP production and phosphorylation of PKA and CREB to express neurogenic and synaptogenic genes [267,269]. Consequently, the activation of ADGRF1 by exogenous treatment of synaptamide alleviates axon degeneration in models of mild TBI and optic nerve crush [270–272]. Further, the ADGRF1-synaptamide interaction also mediates the neuroimmune response. Neuroinflammation triggered by lipopolysaccharide injection is reduced by treatment with synaptamide, which suppresses proinflammatory genes by downregulating NF-*κ*B [269,271,273,274].

Aside from these roles, ADGRF1 is present in brain tissue of patients with glioma. Similar to ADGRD1, ADGRF1 is absent in non-cancerous brain tissue and high ADGRF1 expression is positively correlated with glioma severity, reduced survival rates, and enhanced cell invasion [275]. This suggests that the capability of ADGRF1 to promote neurogenesis may be hijacked to promote the infiltration of glioma cells that occurs in more severe disease cases. Interestingly, epigenome-wide and genome-wide association studies have also associated *ADGRF1* expression with long-term cannabis use [276], as well as chronic shoulder and neck pain in patients with depression [277]. Together, these studies suggest that ADGRF1 could be a prospective therapeutic target for neurodevelopment disorders, axonal degeneration and repair, and glioma.

#### ADGRF2–5

Sparse literature exists for the other members of the ADGRF subfamily, especially regarding functions in the nervous system. Interestingly, ADGRF3 is moderately expressed in human cortical areas, suggesting a potential avenue for future studies (Figure 1). Two studies have found differential expression of ADGRF3 in pancreatic, gastric, small bowel, and duodenal neuroendocrine tumors [278,279], but a definitive role for ADGRF3 has not been discovered. Similarly, the function of ADGRF4 remains unclear, but one study identified the rs1109581 SNP in *ADGRF4* to be associated with Alzheimer’s disease in a non-APOE ε4 carrier [280]. Some work has been performed in ADGRF5, but it appears that its role in the nervous system is limited.

### ADGRGs

#### ADGRG1

ADGRG1 has been extensively studied in the context of development and myelination of the nervous system. Knockout of *Adgrg1* in mice creates neuronal ectopias, resulting in cobblestone lissencephaly-like cortical malformation [281]. Numerous *ADGRG1* mutations in humans have been associated with a specific type of cobblestone lissencephaly, called bilateral frontoparietal polymicrogyria [282–287]. This abnormal development indicates a critical role in ADGRG1 activity during cortical lamination. ADGRG1 follows an anterior-posterior gradient of expression in preplate neurons and is normally expressed by the basal endfeet of radial glial cells [281,288,289]. Loss of *Adgrg1* disrupts the pial basement membrane, leading to neuronal overmigration due to a disruption in localization of radial glial cell endfeet and layer I Cajal-Retzius cells, which are critical in guiding migration [281]. The regulation of cortical development occurs by inhibiting neuronal migration through a binding interaction between ADGRG1 and collagen III [288,290] and localization of ADGRG1 in radial glial cell endfeet is mediated by the activity of MEMO1 [289].

Aberrant ADGRG1 activity also leads to hypomyelination, with a reduction in the number of mature oligodendrocytes due to decreased proliferation of oligodendrocyte precursor cells (OPCs) [291–294]. Disruptions also induce myelination abnormalities in the peripheral nervous system, but do not affect Schwann cell proliferation or differentiation [292]. Interestingly, overexpression of *Adgrg1* increases OPC proliferation and impairs differentiation into mature oligodendrocytes [291]. This suggests that the reduction in OPC proliferation and oligodendrocyte number is due to premature cell cycle exit in OPCs. Indeed, ADGRG1 interacts with microglial transglutaminase-2 to regulate OPC proliferation, which also requires laminin-111 [294]. Knockout of microglial transglutaminase-2 similarly results in hypomyelination resulting from a downregulation of OPC cell cycle progression regulator, CDK2 [294]. These defects in myelination with *Adgrg1* loss can subsequently impair remyelination after injury [294] and induce neuropathy in aging mice [292].

Additional roles for ADGRG1 in the nervous system are still being discovered. Recent evidence suggests that ADGRG1 is involved in synaptic pruning [295], is downregulated in TBI [296], is upregulated with anti-depressant treatment [297], and modulates PV+ interneurons [298]. The ADGRG1 S4 isoform has been found to bind phosphatidylserine on the presynaptic terminal of retinal ganglion cells and does not play a role in OPC proliferation [295]. Loss of *Adgrg1* in microglia leads to impaired ocular dominance columns, increased NMDA receptor-mediated currents, and decreased synaptic pruning by microglia [295,299]. Reduced ADGRG1 activity also upregulates inflammatory cytokines and chemokines in TBI, exacerbating symptoms including motor deficits, short-term memory, spatial memory, lesion volumes, brain water content, BBB damage, and neuronal apoptosis [296]. A clinical study of 424 patients taking the anti-depressant duloxetine revealed that *ADGRG1* was the most upregulated mRNA and was selectively upregulated in patients that responded to the treatment [297]. Further, maternal immune activation reduced levels of glial ADGRG1, and decreased density of PV+ interneurons are observed [298]. This loss of PV+ interneurons has been associated with ASD with mice exhibiting ASD-like behaviors such as anxiety, having no preference for socialization, and increased repetitive behaviors [298]. Conditional knockout of *Adgrg1* in microglia phenocopied PV+ interneuron loss and ASD-like behaviors and upregulation of *Adgrg1* in microglia rescues maternal immune activation-induced deficits [298]. Together, these studies implicate ADGRG1 as a potential therapeutic target for treatment of cortical maldevelopment, myelination disorders, TBI, depression, and ASD.

#### ADGRG6

ADGRG6 plays a role in myelination of the peripheral nervous system. The expression of ADGRG6 initially drives 1:1 sorting of axons by immature Schwann cells via an interaction with Laminin-211 that inhibits cAMP signaling [300–302]. Following maturation of the basal lamina, Laminin-211 is polymerized to increase G_s_ signaling and cAMP expression by promoting activation of the tethered *Stachel* agonist [302,303]. This increase in cAMP drives expression of transcription factors *oct6* and *krox20*, which triggers terminal differential of Schwann cells, expression of myelin-related genes, and initiates myelination [300,304]. Loss of functional ADGRG6 in development leads to delayed sorting of axons, disruption of Remak bundles, and a lack of myelination, leading to nerve and limb defects that are ultimately lethal in mice [301,304,305]. Similarly, human mutations in GPR126 have been associated with lethal arthrogryposis multiplex congenita [306], distal arthrogryposis with patchy neuropathy [307], and lethal congenital contracture syndrome 9 [308]. Myelination defects can be rescued by exogenous expression of cAMP [301,309]. This makes ADGRG6 an interesting therapeutic target as multiple modulators of ADGRG6-mediated cAMP expression have been identified. Aside from Laminin-211, cAMP augmentation by ADGRG6 is also triggered by collagen IV [310] and the prion protein [311]. Meanwhile, collagen VI has been shown to decrease cAMP through G_i_-coupled signaling [312]. Through these pathways, ADGRG6 acts as a key regulator of Schwann cell differentiation.

Despite this extensive role in the initiation of myelination, ADGRG6 has only a limited involvement in the maintenance of myelin. Loss of ADGRG6 in 4-week and 4-month-old mice does not cause defects in Schwann cells or myelination [300]. However, ADGRG6 is essential for repair following nerve injury. ADGRG6 is hypomethylated after injury, increasing expression in activated Schwann cells [313]. This upregulation is critical for remyelination and recruitment of macrophages, both of which are inhibited in *Adgrg6* knockout mice [300]. Further, reinnervation and clustering of acetylcholine receptors in the neuromuscular junction by nonmyelinating terminal Schwann cells also requires ADGRG6 [314]. This repair function is of clinical interest, as activation of ADGRG6 could counteract axonal degeneration in peripheral nerve injuries. Yet, efforts to improve myelin repair by activation of ADGRG6 via the prion protein have been unsuccessful [315,316]. This suggests that Laminin-211 or collagen IV may be better pharmacological targets to mediate ADGRG6-related functions.

There is also evidence for other roles of ADGRG6 in the nervous system. Loss of ADGRG6 leads to impaired vascular development, including defects in the permeability of the blood brain barrier, cortical vasculature, and retinal vasculature [317,318]. Consequently, this leads to embryonic hemorrhage in mice [319] and a meta-analysis of patients with lacunar stroke implicated ADGRG6 in pathogenesis as a key regulator of endothelial dysfunction and pericyte differentiation [320]. In endothelial cells, *Adgrg6* expression closely follows the development of the blood brain barrier, with levels decreasing shortly after establishment [317]. Knockdown of *Adgrg6* inhibits endothelial cell migration and proliferation due to G1/S cell cycle arrest [318]. In this process, Wnt/β-catenin signaling modulates ADGRG6 expression in endothelial cells, which then regulates VEGFR2, STAT5, and GATA2 to promote angiogenesis [318]. Aside from myelination and angiogenesis, ADGRG6 could also play a role in the development of the cerebellum [321] and clustering of axonal sodium channels in the Nodes of Ranvier [322]. Interestingly, a homozygous missense variation in *ADGRG6* has been identified in two patients with severe intellectual disability [323], supporting a larger role for ADGRG6 in the central nervous system.

#### ADGRG2, 3, 4, 5, and 7

Besides ADGRG1 and ADGRG6, there is limited evidence for roles in the nervous system with other members ADGRG subfamily. Work on ADGRG2 has occurred primarily in the context of male infertility while ADGRG3, ADGRG4, and ADGRG5 are involved in the immune system. One study on ADGRG3 has shown that its expression increases with experimental autoimmune encephalomyelitis and that *Adgrg3* knockout mice have exacerbated symptoms, but follow-up studies have yet to be performed [324]. Given that ADGRG2, 3, 4, 5, and 7 are minimally expressed (Figure 1), they may not be directly involved in nervous system function.

### ADGRLs

#### ADGRL1

The functions of ADGRL1–3 in synapse formation and other non-neuronal functions have been extensively reviewed recently [325,326]. Briefly, ADGRL1 was originally discovered as a receptor for black widow venom, or alpha-latrotoxin. Binding of alpha-latrotoxin to ADGRL1 leads to a calcium-independent response [327,328], while binding to neurexin 1α mediates the calcium-dependent response [329,330]. Together, binding to these receptors leads to the formation of Ca^2+^ ionophores [331], secretion of vasopressin and oxytocin [331], release of intracellular Ca^2+^ [331], and excessive neurotransmitter release [327,332,333]. The calcium-independent response through ADGRL1 occurs through modulation of synaptic vesicle fusion machinery, including synaptobrevin, SNAP-25, and Munc13–1 [333]. Typically, autoproteolytic cleavage of ADGRL1 leads to the NTF and CTF acting as independent proteins [334,335]. Binding of alpha-latrotoxin leads to phosphorylation of the CTF, which promotes convergence of the two fragments [334] and induces G protein mediated signaling [328] to regulate K and L-type Ca channels by phospholipase C [328,336]. However, G-protein signaling is not necessarily required for ADGRL1 response to alpha-latrotoxin, suggesting other signaling pathways could be coactive [337]. Dissociation of the two fragments has been shown to reduce alpha-latrotoxin induced neurotransmitter release in the neuromuscular junction [335].

In normal physiology, ADGRL1 is critical in neurodevelopment. Knockout of ADGRL1 leads to embryonic lethality in multiple animal models [338,339], but viability is dependent on genetic background [338]. One study found that loss of *Adgrl1* in mice leads to neurological deficits, including social and sexual interaction defects and hyperactivity [338]. Similarly, ADGRL1 modulates Notch signaling in *C. elegans* through an interaction with Notch ligand LAG-2, leading to morphological and neuronal defects in ADGRL1-deficient animals [340,341]. During axonal migration, ADGRL1 promotes cell adhesion by binding with neurexin 1β [342]. ADGRL1 also directly modulates actin cytoskeleton remodeling through the cofilin pathway, which destabilizes F-actin [343]. Overexpression of ADGRL1 leads to a reduction in cell area, F-actin expression, and F-actin projections, which could lead to the observed neuronal migration defects [343,344].

ADGRL1 is also required for normal synaptic function. Postsynaptic ADGRL1 interacts with presynaptic teneurins and fibronectin-leucine-rich transmembrane proteins (FLRTs) in a trans-synaptic complex to regulate synapse assembly [345–347]. This binding interaction is likely mediated by the lectin-like domain in ADGRL1 and the presence of the β-propeller in teneurin-2 [346,348]. The importance of this interaction has been illustrated in the hippocampus, where ADGRL1 is highly expressed throughout its development [349,350]. Deletion of presynaptic teneurins in the entorhinal cortex led to a loss of synapses onto CA1 of the hippocampus, subiculum, and dentate gyrus [347]. Loss of postsynaptic ADGRL1 phenocopied this effect and rescue in knockouts required the teneurin-binding sites of ADGRL1 [347]. Aside from teneurins, ADGRL1 also binds to neurexin-1β [342] and SHANK [350–352], each of which has unique neurophysiological functions that ADGRL1 may mediate. Interestingly, there is contradictory evidence as to whether ADGRL1 regulates excitatory or inhibitory synapse formation. Teneurins form nanoclusters in excitatory synapses [347] and loss of *Adgrl1* has been found to impair formation of excitatory synapses [338]. However, a different study observed no deficits in excitatory synapse function [353]. Instead, ADGRL1 was required for proper formation and signaling of somatic inhibitory synapses [353]. This could be due to a difference in the genetic background of mice used, but more studies are necessary to resolve this discrepancy. Due to the variety of binding partners of ADGRL1 and the numerous alternative splice forms for each protein [354], it is possible that ADGRL1 could be involved in both excitatory and inhibitory synapse formation depending on specific contexts. Interestingly, *ADGRL1* variants have recently been associated with epilepsy [355], as well as cognitive and language development delay [356], suggesting a critical function in mediating the excitatory / inhibitory balance.

ADGRL1 additionally mediates apoptosis and neuronal sensitization. Overexpression of ADGRL1 triggers neuronal death and is modulated by TAFA2 and Contactin-6 [357,358]. TAFA2 binds ADGRL1 through the lectin-like domain and suppresses apoptosis [357]. Similarly, Contactin-6 can reduce apoptosis induced by overexpression of ADGRL1 [358]. In ischemic conditions, neuronal death in CA1 is significantly higher than in CA3, correlating with upregulation of ADGRL1 in CA1 and downregulation of ADGRL1 in CA3 [359]. Interestingly, ADGRL1 is downregulated in cerebrospinal fluid after TBI [360], suggesting a suppression of apoptosis. Conversely, ADGRL1 has also been found to be upregulated in reactive astrocytes following mechanical brain injury [361]. Further, studies in *Drosophila* have shown that ADGRL1 mediates signal transduction in mechanosensitive and nociceptive neurons [362–364]. ADGRL1 directly modulates electrical activity of mechanoreceptors [363] to modulate relative mechanosensitivity [364] and is required for proper locomotion [363]. Thus, ADGRL1 activity can act as a sliding threshold to alter neuronal sensitization under differential stimulation.

#### ADGRL2

ADGRL2 is a crucial guidance cue for establishing neuronal circuitry. Complete knockout of *Adgrl2* in mice is embryonic lethal [365], but this is likely due to its role in non-neuronal functions [354]. Postsynaptic *Adgrl2* is expressed in specific excitatory synapses of the hippocampus, including in the stratum lacunosum moleculare area of CA1 [366,367], the entorhinal cortex [368,369], presubiculum [368], and parasubiculum [368]. In distal CA1 neurons, ADGRL2 acts as a repulsive receptor for the ligand Teneurin-3, directing axons to the proximal subiculum [370]. Similarly, in proximal CA1 neurons, Teneurin-3 acts as a repulsive receptor for ADGRL2, directing axons to the distal subiculum [370]. Thus, neurons expressing *Adgrl2* or *Teneurin-3* form connections with neurons expressing the same receptor, which is mediated by contact repulsion [369,371]. Further, ADGRL2 GPCR activity and cAMP are required for the establishment of these synapses [372]. Postsynaptic deletion of *Adgrl2* leads to impaired presubiculum *→* medial entorhinal cortex [368,373] and entorhinal cortex *→* stratum lacunosum moleculare [366,367] projections. Synapses in the stratum oriens and stratum radiatum, as well as Schaffer collateral inputs, are unaffected [367,372]. Behaviorally, this results in defective performance in spatiotemporal memory tasks [366,373]. Interestingly, ADGRL2 is modulated in CA1 by oxidative stress [374] and Cajal-Retzius cells [375] and is downregulated in neurodegeneration [354].

ADGRL2 also mediates neuronal connections outside of the hippocampus by similar mechanisms. ADGRL2 is expressed in Purkinje cells of the cerebellum, where it is involved in establishing parallel fiber connections [376]. Intriguingly, loss of ADGRL2 or ADGRL3 alone is not sufficient to observe cerebellar defects, but a double knockout leads to impaired parallel fiber synapses [376]. During human neurodevelopment, ADGRL2 is also expressed in the cortical plate, basal ganglia, pons, and cerebellar cortex [365]. In one clinical case, a heterozygous *ADGRL2* missense variant was associated with extreme microcephaly, absent cortical sulcation, and rhombencephalosynapsis [365], suggesting that ADGRL2 may be involved in development of brain regions outside of the hippocampus and cerebellum. Indeed, deficits in cortical projections have been observed with loss of *Adgrl2* [371,372]. Two recent preprints have also illustrated that Teneurin-3 and ADGRL2 are expressed in opposing concentration gradients that are critical for forming somatosensory maps [377], visual, auditory, basal ganglia, and cerebellar circuits [378], and hippocampus *→* cerebellum projections [378]. Together, these studies demonstrate that ADGRL2 is a key regulator of neuron guidance and that defective *ADGRL2* can lead to lethal neurodevelopmental disorders.

#### ADGRL3

Numerous behavioral phenotypes have been associated with aberrant ADGRL3 activity, suggesting that it is a crucial component of the nervous system. Loss of *Adgrl3* in mice and *adgrl3.1* in zebrafish leads to hyperactivity [379–388], social deficits [380,381], impaired spatial learning and memory [385,386,389], and altered reward-related behaviors [381,382,384,386,387,390]. These behavioral changes share commonalities with attention-deficient hyperactivity disorder (ADHD). Indeed, several studies have found that mutations in human *ADGRL3* are correlated with increased risk of developing ADHD [391–413]. These SNPs do not necessarily cause loss of function, but can instead alter transcription of *ADGRL3* [397,414]. Further, human *ADGRL3* expression has been associated with starvation [397], nicotine exposure [397], and maternal stress [415]. Interestingly, some work has found *ADGRL3* as a determinant of response to ADHD medication methylphenidate [416–418], but this association remains unclear [419]. However, in zebrafish and mice models, various ADHD medications have been shown to attenuate *adgrl3.1* and *Adgrl3* knockout-induced hyperactivity [379,420]. Functionally, patients with *ADGRL3* SNPs have altered electroencephalogram activity when performing a visual Go-NoGo task and made more omission errors, suggesting that executive functions are altered [421]. Beyond ADHD, *ADGRL3* has also been associated with substance use disorder [422], ASD [394,423], chronic migraines [424], ependymoma [425], schizophrenia [426], and Huntington’s disease [426].

Similar to ADGRL1 and ADGRL2, ADGRL3 is involved in glutamatergic synapse development. Postsynaptic ADGRL3 binds to presynaptic Teneurin-2 splice variant Lasso and presynaptic FLRT3 to form a trimeric trans-synaptic protein complex [367,427–429]. The lectin domain of ADGRL3 is required for binding to Teneurin-2 [429] and the olfactomedin domain mediates binding to FLRT3 [430]. Dysfunction of this protein complex or any of its members leads to disruption of excitatory synapses in cultured neurons [427–429,431]. One recent study also found that SARS-CoV-2 viral particles accumulate at the ADGRL3-FLRT3 interface, blocking normal ADGRL3 activity and leading to dysregulated neurons [432]. Synapse modulation by ADGRL3 is dependent on G-protein signaling, specifically through G_αs_ coupling [372,427]. In the hippocampus, deletion *Adgrl3* in mice selectively decreases Schaffer collateral projections to the stratum oriens and stratum radiatum, while deletion of *Adgrl2* selectively decreases entorhinal cortex projections to the stratum lacunosum moleculare [367]. Coincident loss of *Adgrl3* with *Adgrl2* in the cerebellum also leads to decreased parallel fiber synapses on Purkinje cells [376]. In the retina, ADGRL3 regulates horizontal cell synapses [433] and is involved in trans-axonal signaling with Glypican-3 and Teneurin-3 to prune mistargeted retinal projections [434]. Further, loss of *Adgrl3* affects cortical synapses, with decreased synapse density in projections from layer 2/3 *→* layer 5 [435]. Intriguingly, one study has also found that *Adgrl3* and *Flrt3* are downregulated in a hyperactive ADHD mouse model [436], offering a partial explanation for ADHD etiology.

ADGRL3 additionally mediates dopaminergic function throughout the brain. Loss of *Adgrl3* in mice leads to increased levels of dopamine and serotonin in the striatum [384,386,387], due to altered expression of their related transporters [381,383,384,387]. More dramatic effects are observed with knockdown of *adgrl3.1* in zebrafish, which results in loss and mistargeting of dopaminergic neurons in the ventral diencephalon [388] and hyposensitivity to dopamine modulators [437]. Behaviorally, *Adgrl3*-deficient mice had increased reward motivation [390], but impulsive choice is not altered [438]. Functional assays measuring striatal activity in *Adgrl3* knockout mice found dysregulation of dopamine signaling and hyposensitivity to amphetamine treatment [387]. A recent preprint also illustrated greater levels of evoked dopamine release, comparable release capacity to wild-type mice, and defective release during a fixed interval task [439]. Interestingly, in this case, amphetamine treatment rescued dopamine signaling [439]. Together, these studies provide a mechanism for dopamine dysregulation, which could contribute to ADHD development [440].

#### ADGRL4

ADGRL4 has been studied as a proangiogenic factor in gliomas. Interestingly, its extracellular domain differs significantly from ADGRL1–3 and is more similar to the ADGRE subfamily, suggesting that ADGRL4 may be misclassified [441]. In patients with glioma, ADGRL4 upregulation is correlated with tumor progression and decreased survival rates [442–445]. ADGRL4 is selectively expressed by endothelial cells, supporting its involvement in angiogenesis [446,447]. The overexpression of ADGRL4 in glioma is thought to promote tumor angiogenesis and progression by regulating regulates VEGFR2 [448], Notch-1 signaling [449,450] and the JAK/STAT3/HIF-1α axis [444]. In turn, ADGRL4 expression has been found to be regulated by miR-139–5p [451], VEGF-A [443], and TGFβ2 [443]. Excitingly, efforts to target gliomas by silencing ADGRL4 have been successful in murine and cell models. Downregulation of ADGRL4 by antibodies or small interfering RNAs increases survival rates [448–450], inhibits tumor growth [448–452], normalizes vasculature [449,450], prevents disruption of the brain-blood barrier [449], inhibits cell proliferation [451], and increases apoptosis in tumors [450,451,453]. This effect seems to be specific to targeting ADGRL4 as it is not recapitulated by downregulation of VEGFR-2 [448]. Knockdown of ADGRL4 has also been tested in neuroblastoma [454] and retinoblastoma [455], yielding similar positive results. These studies implicate ADGRL4 as an appealing pharmacological target, but more studies need to be conducted into the function and mechanisms of ADGRL4 in gliomas and normal brain physiology.

ADGRL4 has also been preliminarily associated with other neuronal functions. Genomic studies have identified ADGRL4 associations with stroke risk [456], sleep-wake cycle [457], cannabis use disorder [458], oligodendrogliomas [459], and schizophrenia [460]. Further, ADGRL4 may be involved in certain symptoms of multiple sclerosis. In mice with experimental autoimmune encephalomyelitis, ADGRL4 was upregulated throughout the brain, especially in the corpus callosum [461]. This subsequently led to inflammation, disruption of cerebral blood flow, and a leaky BBB [461]. These results suggests that there are various unexplored roles for ADGRL4 in the brain, warranting further studies.

### ADGRV1

#### ADGRV1

ADGRV1, the largest-known cell surface protein, has been linked to the development of Usher syndrome IIC and seizure susceptibility. Numerous mutations in ADGRV1 have been identified in patients with Usher syndrome IIC, which is characterized by moderate to severe hearing loss and vision loss from retinitis pigmentosa [462–476]. Interestingly, ADGRV1 mutations have also been associated with hearing loss, but not Usher syndrome [477–480]. In the developing ear, ADGRV1 participates in Usher protein complexes to establish proper hearing. ADGRV1 is a critical component of ankle links between stereocilia and forms an ankle link protein complex consisting of Usherin 2A, Vezatin, Whirlin, and ADGRV1 [481–484]. This complex has been shown to localize other proteins such as adenylyl cyclase 6 [482] and PDZD7 [485] to modulate hair cell development. Consequently, disruption of *Adgrv1* in mice leads to ear dysfunction and deafness due to loss of outer hair cells and ankle links, improper development of the stereocilia, impaired mechanoelectrical transduction, and disruption in auditory cortex interneuron development [482,484,486–488]. Because of its large size, many ADGRV1 isoforms exist that can participate in differential functions. Distinct variants of ADGRV1 are trafficked to the basal and apical sides of immature hair cells [481]. One such isoform has been found to associate with Clarin-1, CDH23, and PCDH15, and is involved in the formation of hair cell synapses [489]. Together, these studies provide insight into the loss of hearing observed in patients with and without Usher syndrome IIC and implicate ADGRV1 as a therapeutic target for treatment of Usher syndrome IIC-related deafness.

The pathways disrupted by ADGRV1 mutations that lead to retinal pigmentosa in Usher syndrome IIC are less clear. Like in the ear, ADGRV1 interacts with Usherin 2A and Whirlin B in the retina [483,490]. Mouse models with mutant *Adgrv1* have impaired vision and loss of ADGRV1 is observed in the periciliary membrane region and connecting cilium [488,490,491]. This absence of ADGRV1 leads to altered localization and expression Usherin 2A and Whirlin B [488,490]. Subsequently, structural defects in the connecting cilium are observed [488], as well as defective ciliary trafficking of rhodopsin [490]. However, it appears that the morphology of inner and outer photoreceptor segments may be unaffected [492]. Aside from this complex, additional functions of ADGRV1 in the retina have been detected. ADGRV1 can interact with the CCT3 subunit of the TriC / CCT chaperonin complex, which interacts with chaperonin-like BBS proteins [491]. This BBSome protein complex is crucial for ciliary development and protein trafficking but does not explain defective rhodopsin trafficking [493]. These data suggest that ADGRV1 is involved in multiple distinct pathways that are involved in proper visual function.

Deficient ADGRV1 has also been associated with defective focal adhesion. ADGRV1 is crucial for the formation of focal adhesions in astrocytes [494,495], but not their disassembly [495]. Loss of Adgrv1 leads to slower assembly of new focal adhesions due to a disruption in paxillin recruitment [495] and focal adhesion kinase expression [494]. This, in turn, leads to ineffective mechanical-dependent cell migration [494]. Interestingly, loss of ADGRV1 has also been shown to dysregulate autophagy. In human immortalized retinal pigment epithelial cells and Usher Syndrome IIC patient-derived fibroblasts, loss of functional ADGRV1 was associated with increased autophagy [496]. Aberrant autophagy could also potentially contribute to the gradual degeneration of hearing and vision observed in clinical cases of Usher Syndrome IIC.

Aside from its role in Usher syndrome IIC, ADGRV1 has been associated with epilepsy and other neurological dysfunctions. Many human mutations have been identified, primarily in children, in various epileptic phenotypes [497–509]. Low expression of ADGRV1 is also a possible risk factor for epileptogenesis in glioma patients [510]. However, the exact mechanisms behind ADGRV1-related seizures remains elusive. Mouse models with mutant ADGRV1 have been shown to exhibit susceptibility to audiogenic seizures [511,512], suggesting that the mechanisms leading to hearing loss may also lead to aberrant activity in auditory pathways. Considering that ADGRV1 is highly expressed in the developing mouse brain [513] and modulates auditory cortex interneuron development [487], it may also play a role in determining the excitatory / inhibitory balance. Alternatively, defective autophagy could also contribute to the development of seizures. Furthermore, ADGRV1 has been associated with germline telomere length in patients with neuroblastoma [514], opioid dependence risk [515], and megalencephaly-capillary malformation-polymicrogyria syndrome [516]. These studies suggest that there are further neurological functions and isoforms of ADGRV1 yet to be uncovered.

## CONCLUSIONS

The aGPCRs act as diverse modulators of nervous system function. Due to their associations with numerous neurological and psychiatric disorders, as well as numerous isoforms, many are appealing candidates for specific targeting by therapeutics. To achieve this, further studies into many of the aGPCRs are necessary to understand their physiological functions, contributions to etiology of these disorders, and potential as pharmacological targets.

## Figures and Tables

**Table 1.** Members of the aGPCR family. aGPCR alternative name(s) [1], tissue specificity, brain region expression, general expression cluster, brain expression cluster, and single cell type specificity data from the Human Protein Atlas [5].

aGPCR	Alternative name (s)	Tissue Specificity	Highest Brain Region Expression	Expression Cluster	Brain Expression Cluster	Cell Type Specificity
ADGRA1	GPR123	Enriched in brain	Habenula	Brain – neuronal signaling	Brain – neuronal signaling	OPCs, excitatory neurons, horizontal cells, inhibitory neurons
ADGRA2	GPR124	Expressed across many or all tissues	Retina	Connective tissue – extracellular matrix organization	Cerebral cortex – mixed function	Lymphatic endothelial cells, Leydig cells, smooth muscle cells, endometrial stromal cells, peritubular cells, fibroblasts, adipocytes, muller glia cells
ADGRA3	GPR125	Enriched in liver	Choroid plexus	Liver – oxidoreductase activity	Astrocytes – mixed function	Astrocytes, hepatocytes
ADGRB1	Brain-specific Angiogenesis Inhibitor (BAI) 1	Enriched in brain	Precentral gyrus	Astrocytes – astrocyte-neuron interactions	Astrocytes – astrocyte-neuron interactions	OPCs, astrocytes, horizontal cells, excitatory neurons, inhibitory neurons, bipolar cells
ADGRB2	Brain-specific Angiogenesis Inhibitor (BAI) 2	Enriched in brain	Hippocampus	Neurons – mixed function	Neurons – mixed function	Excitatory neurons, horizontal cells, astrocytes, OPCs, inhibitory neurons, bipolar cells
ADGRB3	Brain-specific Angiogenesis Inhibitor (BAI) 3	Enriched in brain	Hippocampus	Neurons – mixed function	Neurons – mixed function	Excitatory neurons, horizontal cells, astrocytes, oligodendrocyte precursor cells, inhibitory neurons, bipolar cells
ADGRC1	Cadherin EGF LAG seven-pass G-type receptor (CELSR) 1	Enhanced in skin	Corpus callosum	Skin – cornification	Brainstem – mixed function	Ciliated cells, glandular and luminal cells, Club cells, Alveolar cells type 1, basal respiratory cells, ionocytes
ADGRC2	Cadherin EGF LAG seven-pass G-type receptor (CELSR) 2	Enhanced in brain and skin	Dentate gyrus	Nonspecific – endocytosis	Neurons – mixed function	Horizontal cells, oligodendrocytes
ADGRC3	Cadherin EGF LAG seven-pass G-type receptor (CELSR) 3	Enhanced in brain, pituitary gland	Flocculonodular lube	Cerebellum – nervous system development	Cerebellum – nervous system development	cone photoreceptor cells, horizontal cells, bipolar cells, rod photoreceptor cells, inhibitory neurons, excitatory neurons
ADGRD1	GPR133	Enhanced in heart muscle	Retina	Smooth muscle tissue – extracellular matrix organization	Subcortical – mixed function	Mesothelial cells, endometrial stromal cells, cardiomyocytes, Sertoli cells, fibroblasts, alveolar cells type 2, microglia
ADGRD2	GPR144	Enhanced in seminal vesicles	Pons	Not detected – no cluster assigned	Not detected – no cluster assigned	Late spermatids, early spermatids
ADGRE1	EGF-like module-containing mucin-like hormone receptor-like (EMR) 1; F4/80	Enhanced in bone marrow and lymphoid tissue	Corpus callosum	Lymphoid tissue – immune response	Macrophages and microglia – immune response	Monocytes, Kupffer cells
ADGRE2	EGF-like module-containing mucin-like hormone receptor-like (EMR) 2	Enhanced in lymphoid tissue	Retina	Lymphoid tissue – immune response	Non-specific – immune response	Monocytes, granulocytes, macrophages, Kupffer cells
ADGRE3	EGF-like module-containing mucin-like hormone receptor-like (EMR) 3	Enhanced in bone marrow and lymphoid tissue	Area parastriata, superior	Lymphoid tissue – immune response	Non-specific – vasculature	Monocytes, macrophages, mucus glandular cells, microglia
ADGRE4	EGF-like module-containing mucin-like hormone receptor-like (EMR) 4	No information available	No information available	No information available	No information available	No information available
ADGRE5	CD97	Enhanced in bone marrow	White matter	Lymphoid tissue and bone marrow – innate immune response	White matter – signal transduction	Monocytes, NK-cells, T-cells, dendritic cells
ADGRF1	GPR110	Enriched in esophagus, kidney, urinary bladder	Not detected	Epithelium – extracellular exosomes	Not detected – no cluster assigned	Ionocytes, Club cells, collecting duct cells, ciliated cells, basal respiratory cells, glandular cells, luminal cells
ADGRF2	GPR111	No information available	No information available	No information available	No information available	No information available
ADGRF3	GPR113	Enhanced in pancreas	Retina	Stomach – proteolysis	Nonspecific – transcription	Late spermatids, astrocytes, early spermatids, oligodendrocytes, microglia
ADGRF4	GPR115	Enhanced in esophagus and skin	Arcuate nucleus	Skin – cornification	Hypothalamus – neuropeptide signaling	Suprabasal keratinocytes, extravillous trophoblasts, squamous epithelial cells, syncytiotrophoblasts, distal enterocytes, basal keratinocytes
ADGRF5	GPR116; Ig-Hepta	Enhanced in lung	Retina	Adipose tissue – mixed function	Endothelial cells – vasculature	Adipocytes, alveolar cells type 2, endothelial cells, alveolar cells type 1, microglia
ADGRG1	GPR56	Enhanced in thyroid gland	Amygdala	Brain – neuronal signaling	Brain – neuronal signaling	Cytotrophoblasts, NK-cells, syncytiotrophoblasts, melanocytes
ADGRG2	GPR64; HE6	Enriched in epididymis	Pituitary gland	Epididymis – male reproductive secretion	Forebrain – mixed function	Serous glandular cells, secretory cells, prostatic glandular cells, mucus glandular cells, gastric mucus-secreting cells, OPCs
ADGRG3	GPR97	Enriched in bone marrow	Area parastriata, superior	Bone marrow – innate immune response	Nonspecific – vasculature	Lymphatic endothelial cells
ADGRG4	GPR112	Enhanced in fallopian tube, intestine, and retina	Retina	Intestine – digestion	Not detected – no cluster assigned	Enteroendocrine cells, Paneth cells
ADGRG5	GPR114	Enriched in intestine and lymphoid tissue	Thalamus	Lymphoid tissue – immune response	Hindbrain – mixed function	Dendritic cells, NK-cells, plasma cells, microglia, B-cells, T-cells
ADGRG6	GPR126	Enhanced in liver and placenta	Retina	Liver – plasma proteins	Subcortical – mixed function	Hepatocytes, suprabasal keratinocytes
ADGRG7	GPR128	Enriched in intestine and liver	Caudate nucleus	Liver and intestine – lipid metabolism	Not detected – no cluster assigned	Proximal enterocytes, hepatocytes, Paneth cells, intestinal goblet cells
ADGRL1	Latrophilin-1, Calcium-independent receptor of α-Latrotoxin (CIRL) 1, CL-1	Enhanced in brain	Retrosplenial cortex	Astrocytes and cerebellum – nervous system development	Astrocytes and cerebellum – nervous system development	Horizontal cells, excitatory neurons, bipolar cells, inhibitory neurons
ADGRL2	Latrophilin-2, Calcium-independent receptor of α-Latrotoxin (CIRL) 2, CL-2	Expressed across many or all tissues	Cerebral cortex	Adipose tissue – mixed function	Neurons - mixed	Excitatory neurons, inhibitory neurons
ADGRL3	Latrophilin-3; Calcium-independent receptor of α-Latrotoxin (CIRL) 3, CL-3	Enhanced in brain	Ventromedial nucleus	Brain – neuronal signaling	Brain – neuronal signaling	OPCs, inhibitory neurons, excitatory neurons, astrocytes, oligodendrocytes
ADGRL4	EGF, latrophilin, and 7TM domain–containing protein 1 (ELTD1)	Enhanced in adipose tissue	Pituitary gland	Adipose tissue – mixed function	Endothelial cells – vasculature	Adipocytes, endothelial cells
ADGRV1	Very-large G protein-coupled receptor 1; GPR98	Enriched in adrenal gland	Pituitary gland	Adrenal gland – steroid metabolism	Neurons – mixed function	Astrocytes

**Table 2.** Structural domains and motifs in aGPCR subfamilies. aGPCR subfamily, binding domains, and other prominent NTF and CTF motifs and structures identified.

aGPCR subfamily	Binding domain(s)	Other significant NTF structures	CTF motifs and structures
ADGRA [21]	LRR* Ig*	GAIN^†^ HBD* RGD*	PBM
ADGRB [22]	CUB* TSR	GAIN HBD RGD*	PRS PBM
ADGRC [23]	Cad EGF LAG	GAIN	PRS
ADGRD [24]	PDB*	GAIN	PBM
ADGRE [25,26]	EGF	GAIN	PBM
ADGRF [27]	EGF* Ig*	GAIN* HBD* SEA*	
ADGRG [28,29]	CUB* PBD*	HBD* GAIN RGD*	PBM
ADGRL [24]	GBL OLMD	GAIN HBD*	PRS PBM
ADGRV [30,31]	EAR PDB	GAIN Calxβ	PBM

Structural motifs denoted by an asterisk (*) are not present in all protein subfamily members.

† ADGRA1/GPR123 is the only aGPCR lacking the GAIN domain.

**Table 3.** aGPCR genomic locations and associated neurological or psychiatric disorders. Genomic locations were acquired from the National Center for Biotechnology Information Genome primary assembly GRCh38.p14 [65]. Associated neurological or psychiatric disorders in humans that have been identified by genome-wide associated studies or as clinical case reports.

aGPCR	Genomic Location (GRCh38.p14)	Associated Neurological or Psychiatric Disorders
*ADGRA1*	NC_000010.11:133087924–133131675 (+)	Expression associated with better prognosis for glioma (long non-coding RNA variant *ADGRA1-AS1*) [71]. No associations with neurological or psychiatric disorders found for *ADGRA1*.
*ADGRA2*	NC_000008.11:37796883–37844896 (+)	Mutations associated with polymicrogyria [85]. Mutations associated with malformation of cerebellum, spinal cord, and cerebral cortex [86]. Variants with reduced risk of brain arteriovenous malformation [87]. Associated with development of brain metastases in patients with lung adenocarcinoma [95]. Mutations associated with rectal neuroendocrine carcinomas [96].
*ADGRA3*	NC_000004.12:22387376–22516066 (−)	No associations with neurological or psychiatric disorders found.
*ADGRB1*	NC_000008.11:142449649–142545007 (+)	Mutations associated with autism spectrum disorder [107]. Downregulated in medulloblastoma, glioblastoma, astrocytoma, and lung adenocarcinoma brain metastases [109–115].
*ADGRB2*	NC_000001.11:31727117–31764340 (−)	Expression associated with depression [130]. Expression associated with neuroticism [131]. Expression associated with decreased educational attainment [132].
*ADGRB3*	NC_000006.12:68635282–69389506 (+)	Expression associated with anxious temperament [152]. Expression associated with taste perception degeneration in Alzheimer’s disease [153]. Expression associated with Chiari Malformation Type I [154]. Expression associated with disorganized symptoms of schizophrenia [155, 156]. Expression associated with multiple sclerosis [157]. Expression associated with predisposition to substance use disorders [158]. Expression associated with cerebral and cerebellar atrophy [159]. Expression associated with intellectual disability [159]. Expression associated with major depressive disorder [155]. Expression associated with autism spectrum disorder [160]. Downregulated in glioma [150].
*ADGRC1*	NC_000022.11:46361174–46537620 (−)	Mutations associated with neural tube defects and brain malformations [162–171]. Mutations associated with partial epilepsy of childhood [172]. Mutations associated with ischemic stroke [173–175]. Mutations associated with spina bifida [176]. Mutations associated with glaucoma [177]. Mutations associated with familial strabismus [178]. Mutations associated with Phelan-McDermid syndrome [179]. Mutations associated with Parkinson’s disease [180]. Expression associated with glioma [181]. Expression associated with cerebral ischemic injury [182]. Expression associated with child behavioral issues [183].
*ADGRC2*	NC_000001.11:109249539–109275751 (+)	Mutations associated with neural tube defects [170]. Mutations associated with idiopathic scoliosis [198]. Mutations associated with Joubert syndrome [199, 200]. Mutations associated with epilepsy [201]. Mutations associated with Alzheimer’s disease [202].
*ADGRC3*	NC_000003.12:48636463–48662886 (−)	Mutations associated with Tourette’s syndrome [217–220]. Mutations associated with epilepsy [221, 222]. Mutations associated with Rubinstein-Taybi syndrome [223]. Mutations associated with schizophrenia [223]. Mutations associated with oral squamous cell carcinoma perineural invasion [224]. Mutations associated with migraine [225]. Mutations associated with stroke [225]. Mutations associated with central hypotonia [226]. Mutations associated with neuroendocrine cancers [227–229].
*ADGRD1*	NC_000012.12:130953907–131141469 (+)	Expression associated with glioma severity [255, 257, 258].
*ADGRD2*	NC_000009.12:124450451–124478580 (+)	No associations with neurological or psychiatric disorders found.
*ADGRE1*	NC_000019.10:6887579–6940450 (+)	Mutations associated with increased risk of complex malaria-associated seizures in children with falciparum malaria [263]. Mutations associated with high-risk neuroblastoma [264].
*ADGRE2*	NC_000019.10:14724171–14778560 (−)	No associations with neurological or psychiatric disorders found.
*ADGRE3*	NC_000019.10:14600117–14674844 (−)	No associations with neurological or psychiatric disorders found.
*ADGRE4*	NC_000019.10:6950758–6997851 (−)	No associations with neurological or psychiatric disorders found.
*ADGRE5*	NC_000019.10:14381444–14408723 (+)	Expression associated with invasion of glioma cells [265, 266].
*ADGRF1*	NC_000006.12:46997708–47042332 (−)	Expression associated with glioma severity [275]. Expression associated with long-term cannabis use [276]. Expression associated with chronic shoulder and neck pain in patients with depression [277].
*ADGRF2*	NC_000006.12:47656472–47697794 (+)	No associations with neurological or psychiatric disorders found.
*ADGRF3*	NC_000002.12:26308173–26346789 (−)	Expression associated with pancreatic, gastric, small bowel, and duodenal neuroendocrine tumors [278, 279].
*ADRGF4*	NC_000006.12:47698580–47722014 (+)	Mutation associated with Alzheimer’s disease in a non APOE ε4 carrier [280].
*ADGRF5*	NC_000006.12:46852522–46954939 (−)	No associations with neurological or psychiatric disorders found.
*ADGRG1*	NC_000016.10:57619738–57665567 (+)	Mutations associated with bilateral frontoparietal polymicrogyria [282–287]. Downregulated with traumatic brain injury [296]. Upregulated with anti-depressant treatment [297].
*ADGRG2*	NC_000023.11:18989307–19122956 (−)	No associations with neurological or psychiatric disorders found.
*ADGRG3*	NC_000016.10:57665629–57689378 (+)	No associations with neurological or psychiatric disorders found.
*ADGRG4*	NC_000023.11:136300963–136416890 (+)	No associations with neurological or psychiatric disorders found.
*ADGRG5*	NC_000016.10:57529073–57577189 (+)	No associations with neurological or psychiatric disorders found.
*ADGRG6*	NC_000006.12:142302007–142446261 (+)	Mutations associated with lethal arthrogryposis multiplex congenita [306]. Mutations associated with distal arthrogryposis with patch neuropathy [307]. Mutations associated with lethal congenital contracture syndrome 9 [308]. Expression associated with lacunar stroke [320]. Mutations associated with severe intellectual disability [323].
*ADGRG7*	NC_000003.12:100609601–100695479 (+)	No associations with neurological or psychiatric disorders found.
*ADGRL1*	NC_000019.10:14147743–14206169 (−)	Mutations associated with epilepsy [355]. Mutations associated with cognitive and language development delay [356].
*ADGRL2*	NC_000001.11:81306132–81993932 (+)	Mutation associated with extreme microcephaly, absent cortical sulcation, and rhombencephalosynapsis [365].
*ADGRL3*	NC_000004.12:61200326–62078335 (+)	Mutations associated with attention-deficit hyperactivity disorder [391–413]. Expression associated with starvation [397]. Expression associated with nicotine exposure [397]. Expression associated with maternal stress [415]. Expression associated with substances use disorder [422]. Mutations associated with autism spectrum disorder [394, 423]. Mutations associated with chronic migraines [424]. Mutations associated with ependymoma [425]. Mutations associated with schizophrenia [426]. Mutations associated with Huntington’s disease [426].
*ADGRL4*	NC_000001.11:78889764–79006730 (−)	Expression associated with glioma progression [442–445]. Expression associated with stroke risk [456]. Expression associated with sleep-wake cycle [457]. Expression associated with cannabis use disorder [458]. Expression associated with oligodendrogliomas [459]. Mutations associated with schizophrenia [460].
*ADGRV1*	NC_000005.10:90558797–91164437 (+)	Mutations associated with development of Usher Syndrome IIC [462–476]. Mutations associated with hearing loss [477–480]. Mutations associated with seizure susceptibility [497–509]. Expression associated with epileptogenesis in glioma patients [510]. Expression associated with neuroblastoma [514]. Expression associated with opioid dependence risk [515]. Mutations associated with megalencephaly-capillary malformation polymicrogyria syndrome [516].

## Data Availability

The dataset analyzed in the study can be found at the Human Protein Atlas (https://www.proteinatlas.org/), the Allen Brain Atlas Human Multiple Cortical Areas SMART-seq dataset (https://portal.brain-map.org/atlases-and-data/rnaseq/human-multiple-cortical-areas-smart-seq), and the National Center for Biotechnology Information Genome assembly GRCh38.p14 (https://www.ncbi.nlm.nih.gov/datasets/genome/GCF_000001405.40/)

## ABBREVIATIONS

aGPCR: adhesion G protein-coupled receptor
GPCR: G protein-coupled receptor
7TM: seven-transmembrane pass
NTF: N-terminal fragment
GAIN: GPCR autoproteolysis-inducing domain
CTF: C-terminal fragment
cryo-EM: cryogenic electron microscopy
LRR: leucine-rich repeat
Ig: immunoglobulin-like
EGF: epidermal growth factor-like
EAR: epilepsy associated repeat
CUB: complement C1r/C1s, Uegf, Bmp1 domain
TSR: type-1 thrombospondin repeat
GBL: galactose-binding lectin domain
Cad: Cadherin repeat
HBD: hormone binding domain
GPS: GPCR proteolysis site
Lam: laminin domain
PLL: pentraxin/laminin/neurexin/sex-hormone-binding-globulin-like domain
RBL: rhamnose-binding lectin
OLMD: olfactomedin-like domain
LAG: laminin G-like
EAR: epilepsy-associated repeat
PDB: pentraxin-binding domain
SEA: sea urchin sperm protein/enterokinase/agrin module
Calxβ: Calx-beta motif
PBM: PDZ binding motif
RGD: Arg-Gly-Asp motif
PRS: proline-rich sequence
ECL: extracellular loops
ICL: intracellular loops
PV+: paralvbumin-positive
BBB: blood-brain barrier
TBI: traumatic brain injury
ASD: autism spectrum disorder
MDB2: methyl-CpG binding domain protein 2
EZH2: Enhancer of zeste homolog 2
C1QL: component complement 1, Q subcomponent-like
SNP: single nucleotide polymorphism
OPC: oligodendrocyte precursor cell
FLRT: fibronectin-leucine-rich transmembrane protein
ADHD: attention-deficient hyperactivity disorder
